# Intracardiac migration of distal catheter—a rare complication of VP shunt insertion: case report and literature review

**DOI:** 10.1007/s00381-023-06187-6

**Published:** 2023-10-19

**Authors:** Ella Hobbs, Dominic N. P. Thompson, Nagarajan Muthialu, Adikarige Haritha Dulanka Silva

**Affiliations:** 1 School of Medicine, Faculty of Medical Sciences, Newcastle University, Newcastle upon Tyne, UK; 2 Department of Neurosurgery, Great Ormond Street Hospital for Children, London, UK; 3 Great Ormond Street Institute of Child Health, University College London, London, UK; 4 Department of Cardiothoracic Surgery, Great Ormond Street Hospital for Children, London, UK

**Keywords:** Ventriculoperitoneal shunt, Distal migration, Heart, Interventional radiology, Minimally invasive

## Abstract

Intracardiac migration is a rare complication of ventriculoperitoneal shunt insertion. Only 15 cases have been reported, 7 of which were paediatric cases, treated with techniques including interventional radiography, open thoracotomies and direct extraction through the initial shunt incision. The authors report the youngest case of intracardiac shunt migration complicated by significant coiling and knotting within the cardiac chambers and pulmonary vasculature. Migration likely began when the SVC was pierced during initial shunt placement and progressed due to negative intrathoracic pressure. Extrusion was achieved combining thoracoscopic endoscopy, interventional fluoroscopy screening and a posterolateral neck incision with uncoiling of the shunt via a Seldinger guide wire. This offered a minimally invasive solution with rapid post-operative recovery.

## Introduction

Ventriculoperitoneal shunt (VPS) insertion is the most commonly performed paediatric neurosurgical procedure to treat hydrocephalus [1], with 3000–3500 shunt operations carried out across UK annually [2] (13% of all neurosurgical procedures) [3].

VPS complications are common, with approximately 20–40% failing within the first year of placement [1, 4]; many patients undergo multiple revisions throughout their lifetime [1, 3]. Common causes of VPS malfunction are obstruction, infection (3–15%), mechanical shunt failure and migration [4].

VPS migration is relatively uncommon, reported at ~ 1 in 1000 cases [5]. Intestinal perforation is the commonest site, more frequent in children than adults (80.6% vs 19.4%), followed by scrotal and abdominal wall migration [5]. Rarer sites include anal/vaginal extrusion [6]. Intracardiac displacement of the distal catheter is one of the rarest of all VPS complications, with only 7 paediatric cases reported. This life-threatening complication requires meticulous planning and execution of treatment strategies including open thoracotomy, through shunt (retro-auricular) or additional incisions (trans-femoral, cervical) (Table 1) [7].

**Table 1:** Literature review of recorded intracardiac ventriculoperitoneal shunt migrations

Authors and year	Age of patient at time of extraction procedure	Extraction procedure	Complications noted
Morell et al. (1994) [8]	12	Initial attempt via shunt (retro-auricular) incision. Eventual removal achieved through staged IR/fluoroscopic guided snare via femoral vein	Attempt via shunt (retro-auricular) incision complicated by traction induced arrythmias
Kang et al. (1996) [9]	12	Open thoracotomy/sternotomy	None
Frazier et al. (2002) [10]	14	Shunt (occipital) incision and subxiphoid incision	None
Fewel and Garton (2004) [11]	16	Shunt (retro-auricular) incision with IR/fluoroscopic guided visualisation	None
Rizk et al. (2009) [12], case 1	6	Shunt (retro-auricular) incision	None
Rizk et al. (2009) [12], case 2	6	Shunt (retro-auricular) incision	None
Ruggiero et al. (2010) [13]	14	Cervical neck incision with IR/fluoroscopic guided visualisation	None
Hobbs et al. (2023) (Current study)	4	Posterolateral neck incision with video-assisted thoracoscopy (VATS) and IR/fluoroscopic guided visualisation Uncoiling of the distal shunt by Seldinger guide wire insertion	None

We present the youngest reported case of intracardiac distal catheter migration: a 4-year-old boy treated using combined fluoroscopic-guided transluminal and surgical video-assisted thoracoscopic surgical (VATS) approach for distal catheter extraction.

## Case report

**Fig. 1:**
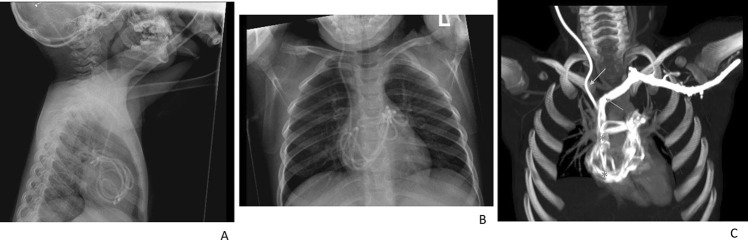
**A**, **B** Presenting lateral and AP chest X-rays demonstrating intracardiac positioning and coiling of the distal VP shunt tubing (red stars). **C** 3D cardiac CT film with contrast demonstrating intracardiac shunt positioning with SVC involvement (yellow arrow), coiling in the right atrium and ventricle (red stars) and extension into both pulmonary arteries (green arrows)

**Fig. 2:**
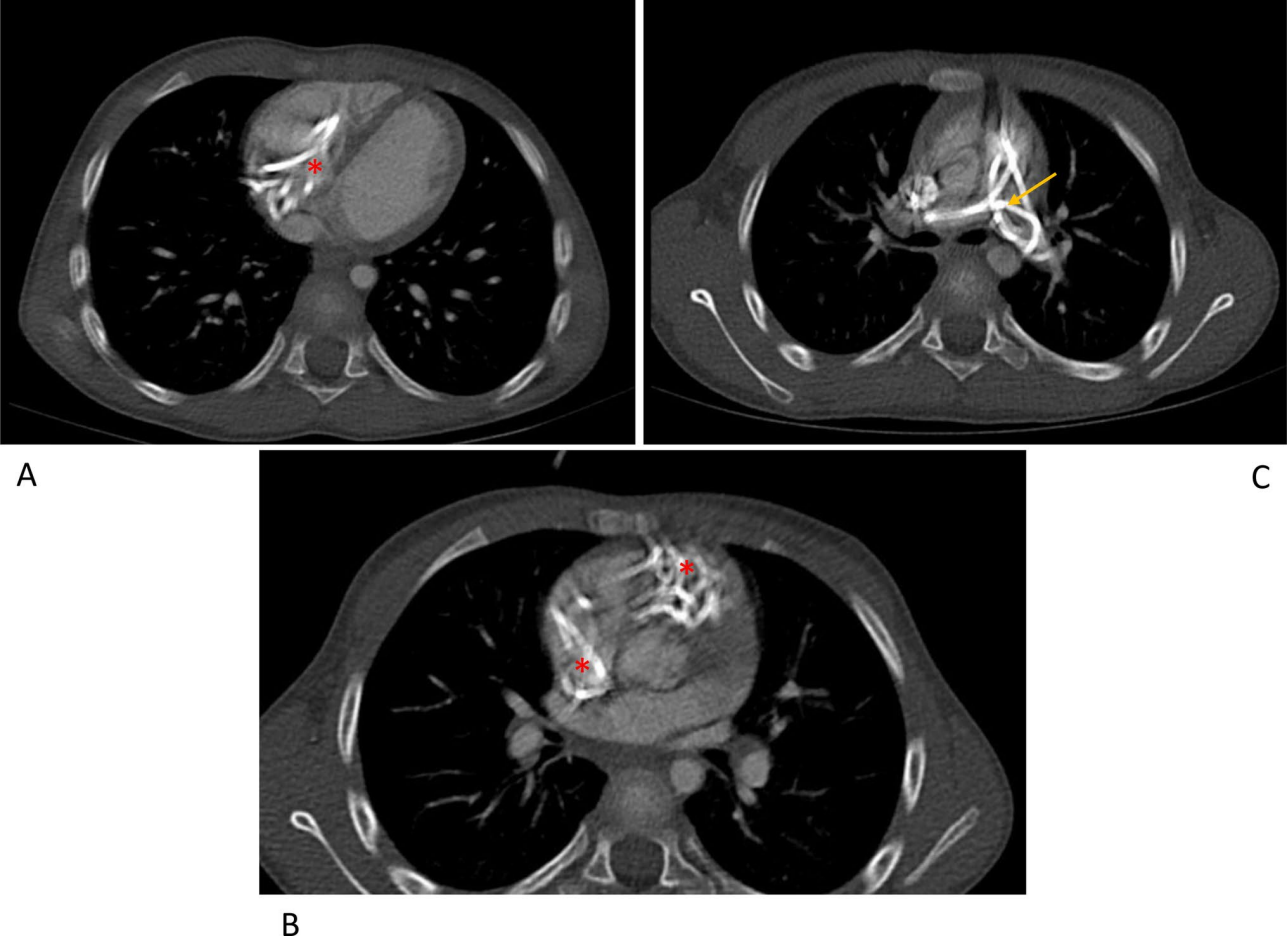
Axial thoracic CT scan images demonstrating extent of shunt coiling and knotting within the atrium and ventricles (**A**, **B**) (red stars) and pulmonary arteries (**C**) (yellow arrow) indicating the significant complication risks of removing the distal shunt via direct extraction

A 4-year-old boy with Chiari type 1 malformation underwent foramen magnum decompression (FMD) at another institution. He developed hydrocephalus and underwent VPS placement. Insertion was reported as challenging with back-bleeding through shunt-valve pocket during cranial-to-caudal/peritoneal tunnelling. Post-operatively significant neck pain, chest wall and abdominal bruising were reported. Three months later, he presented with abdominal and chest pain. Lateral and AP X-rays (Fig. 1A, B) suggested intracardiac migration of the distal catheter, confirmed with chest/cardiac CT (Fig. 1C), indicating that the distal catheter had entered the superior vena cava (SVC) with coiling, and knotting within the right atrium and ventricle, extending into pulmonary arteries (Fig. 2A–C). He was referred to our institution.

### Surgical technique

Multi-disciplinary discussion was held between neurosurgery, cardiac surgery, interventional radiology (IR) and cardiology. Owing to significant risks of thrombi, arrythmias and mechanical cardiac damage, catheter removal was indicated. Due to shunt catheter knotting and transgression through multiple valves and chambers, simple retraction endovascularly or via shunt incision carried unacceptable risk.

Direct catheter retrieval through open sternotomy and cardiac bypass was considered. However, as the catheter entry point was via the SVC, a less-invasive approach using VATS to visualise entry point was devised with IR/fluoroscopic biplanar visualisation. This included cardiac and neurosurgical anaesthetic expertise with echocardiography and arrhythmia monitoring. Open thoracotomy conversion was prepared for if required.

**Fig. 3:**
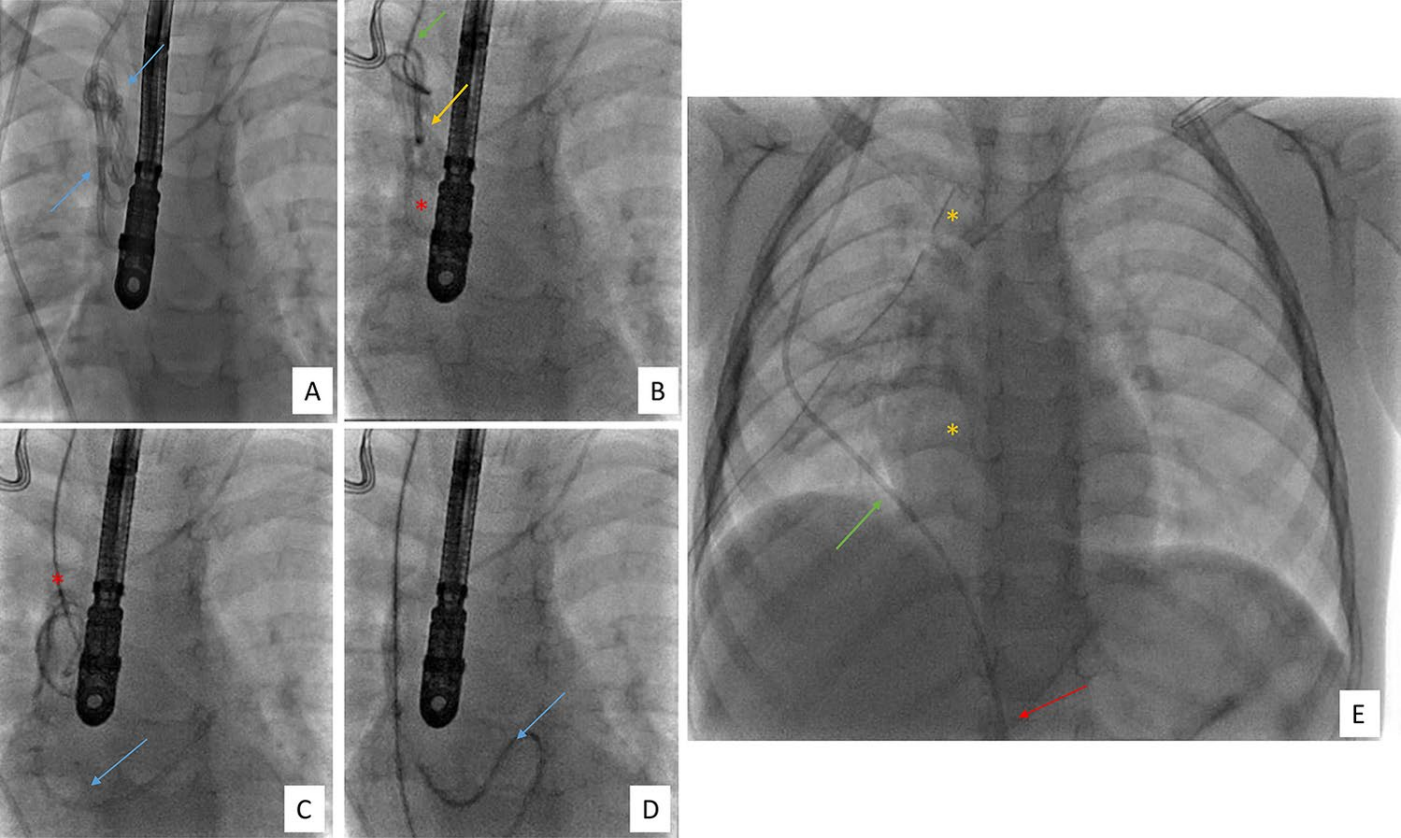
Intraoperative angiographies demonstrating progression of extraction procedure. **A** Angiography taken at beginning of the procedure showing extent of shunt knotting in the right SVC (blue arrows). **B** Demonstrating guide wire insertion (green arrow) through the SVC (yellow arrow) into the right atrium (red star). **C** Guide wire (red star) in process of uncoiling distal shunt with shunt still positioned within the right ventricle (blue arrow). **D** Complete uncoiling of distal shunt before extraction from the right ventricle (blue arrow). **E** Thoracic angiography taken at the end of procedure demonstrating full extraction out of the heart and SVC (yellow stars) and correct positioning of the shunt within the peritoneal cavity (red arrow) crossing the diaphragm (green arrow)

The distal catheter was identified in the neck via a posterolateral-supraclavicular neck incision. It was tunnelled deep to the clavicle rather than superficial. The proximal part was exteriorised as an external ventricular drain (EVD). VAT pleural cavity ports were sited. The mediastinal pleura anterior to the SVC, above level of SVC-azygos vein junction, was dissected. Shunt tubing was seen through the wall of the SVC and atrium, but no obvious entry point could be identified. Under thoracoscopic and IR/fluoroscopic visualisation, an attempt was made to extract the catheter through the neck incision, but obvious resistance was encountered after 40 cm. Fluoroscopy confirmed extraction from the pulmonary arteries and right ventricle but knotting within the right atrium and SVC. A Seldinger guidewire was passed into the distal catheter allowing progressive uncoiling of the catheter and controlled extraction (Fig. 3). Via thoracoscopy, direct pressure was applied at the SVC before temporary chest drain sited.

There were no acute post-operative complications, and a new distal peritoneal catheter was uneventfully inserted 3 days later.

## Discussion

Intracardiac catheter migration is a rare but potentially life-threatening complication of VPS. The most likely mechanism was perforation of a large neck vessel (internal jugular) or chest (SVC) at time of tunnelling, creating a ‘through-and-through’ injury. The catheter would have passed in and out of the vessel during cranial-to-caudal tunnelling. The sub-clavicular catheter location also likely increases risk of perforation not only of a vessel but the pleura. It is possible that catheter ‘tethering’ at the perforation site, combined with negative intrathoracic pressure, led to migration from the abdomen into the heart via the vessel perforation. The history of difficult tunnelling, bleeding, sub-clavicular passage (in itself a risk factor for pleural perforation) and immediate post-operative chest wall bruising was in retrospect significant, although it is a moot point whether post-operative X-rays would have indicated the complication. Identification of excessive backflow bleeding should raise concern about vessel transgression, and one should consider catheter removal, pressure to control bleeding and tunnelling a new catheter via alternative trajectory.

Whilst ventriculoatrial shunts have been successful as an alternative to VPS, the amount of intracardiac tubing in this case presented increased risk of complications including thrombus, pulmonary embolism and heart valve/myocardial injury. Echocardiography is essential preoperatively to identify thrombus formation which can affect management options [12].

Ten of 15 previously reported cases of intracardiac catheter migration were treated with either intravascular fluoroscopic retraction or direct retraction via existing shunt (retro-auricular) or additional (cervical/transfemoral) incisions [8, 13–19]. However, these techniques can be difficult with intracardiac knotting or coiling of tubing within multiple chambers risking mechanical damage to the valve leaflets and myocardium [7]. Furthermore, traction on shunt tubing during removal can induce arrhythmias, requiring a multistage procedure and use of radiologically guided snares to manage [8].

One report described a similar case treated by open thoracotomy and direct extraction [13]. Although no complications were reported, the extended recovery associated with such a procedure and risks of bypass and heparinisation is significant. Our approach is the first reported utilisation of VATS and Seldinger wires to uncoil the distal catheter in as minimally invasive an approach as possible. Meticulous preparation, planning and provision both anaesthetically and surgically for rapid conversion to open sternotomy in case of an intracardiac or vessel injury is essential.

## Conclusion

Intracardiac distal catheter migration is an extremely rare, life threatening complication of VPS insertion. In cases with a difficult distal catheter placement complicated with bleeding/exceptional chest wall bruising, a high degree of vigilance and surveillance is required. Preoperative planning should include shunt X-rays, chest CT and echocardiograms. Multidisciplinary expertise with IR and cardiac techniques facilitated a successfully minimally invasive solution to this complication.

## References

[1] Paff M, Alexandru-Abrams D, Muhonen M, Loudon W (2018). Ventriculoperitoneal shunt complications: a review. Interdiscip Neurosurg.

[2] Richards HK, Seeley HM, Pickard JD (2009). Efficacy of antibiotic-impregnated shunt catheters in reducing shunt infection: data from the United Kingdom Shunt Registry. J Neurosurg Pediatr.

[3] Dakurah TK, Adams F, Iddrissu M, Wepeba GK, Akoto H, Bankah P (2016). Management of hydrocephalus with ventriculoperitoneal shunts: review of 109 cases of children. World Neurosurg.

[4] Hanak BW, Bonow RH, Harris CA, Browd SR (2017). Cerebrospinal fluid shunting complications in children. Pediatr Neurosurg.

[5] Harischandra L, Sharma A, Chatterjee S (2019). Shunt migration in ventriculoperitoneal shunting: a comprehensive review of literature. Neurol India.

[6] Teegala R, Kota LP (2012). Unusual complications of ventriculo peritoneal shunt surgery. Journal of neurosciences in rural practice.

[7] Wei Q, Qi S, Peng Y, Fan J, Lu Y (2012). Unusual complications and mechanism: migration of the distal catheter into the heart—report of two cases and review of the literature. Childs Nerv Syst.

[8] Morell RC, Bell WO, Hertz GE, D'Souza V (1994). Migration of a ventriculoperitoneal shunt into the pulmonary artery. J Neurosurg Anesthesiol.

[9] Kang JK, Jeun SS, Chung DS, Lee IW, Sung WH (1996). Unusual proximal migration of ventriculoperitoneal shunt into the heart. Childs Nerv Syst.

[10] Frazier JL, Wang PP, Patel SH, Benson JE, Cameron DE, Hoon AH Jr et al (2002) Unusual migration of the distal catheter of a ventriculoperitoneal shunt into the heart: case report. Neurosurgery 51(3):819–22; discussion 22 12188965

[11] Fewel ME, Garton HJ (2004). Migration of distal ventriculoperitoneal shunt catheter into the heart: case report and review of the literature. J Neurosurg Pediatr.

[12] Rizk E, Dias MS, Verbrugge J, Boop FA (2009). Intracardiac migration of a distal shunt catheter: an unusual complication of ventricular shunts: report of 2 cases. J Neurosurg Pediatr.

[13] Ruggiero C, Spennato P, De Paulis D, Aliberti F, Cinalli G (2010). Intracardiac migration of the distal catheter of ventriculoperitoneal shunt: a case report. Childs Nerv Syst.

[14] Chong JY, Kim JM, Cho DC, Kim CH (2008). Upward migration of distal ventriculoperitoneal shunt catheter into the heart: case report. Journal of Korean Neurosurgical Society.

[15] Hermann EJ, Zimmermann M, Marquardt G (2009) Ventriculoperitoneal shunt migration into the pulmonary artery. Acta Neurochir 151:647–52 10.1007/s00701-009-0282-9 19350205

[16] Imamura H, Nomura M (2002). Migration of ventriculoperitoneal shunt into the heart—case report. Neurol Med Chir.

[17] Kim MS, Oh C-W, Hur JW, Lee J-W, Lee HK (2005). Migration of the distal catheter of a ventriculoperitoneal shunt into the heart: case report. Surg Neurol.

[18] Kubo S, Takimoto H, Takakura S, Iwaisako K, Yamanaka K, Hosoi K (2002). Peritoneal shunt migration into the pulmonary artery—case report. Neurol Med Chir.

[19] Nguyen HS, Turner M, Butty SD, Cohen-Gadol AA (2010). Migration of a distal shunt catheter into the heart and pulmonary artery: report of a case and review of the literature. Childs Nerv Syst.

